# Generation of reactive oxygen species by human mesothelioma cells

**DOI:** 10.1038/sj.bjc.6690316

**Published:** 1999-04

**Authors:** K Kahlos, S Pitkänen, I Hassinen, K Linnainmaa, V L Kinnula

**Affiliations:** Departments of Internal Medicine, andMedical Biochemistry, University of Oulu, Kajaanintie 50A, FIN-90220 Oulu, Finland; Department of Dermatology, University of Helsinki, Meilahdentie 2, FIN-00250, Helsinki, Finland; Finnish Institute of Occupational Health, Topeliuksenkatu 41A, FIN-00250 Helsinki, Finland

**Keywords:** mesothelioma, reactive oxygen species, oxidant, antioxidant, superoxide dismutase, mitochondria

## Abstract

Malignant mesothelioma cells contain elevated levels of manganese superoxide dismutase (MnSOD) and are highly resistant to oxidants compared to non-malignant mesothelial cells. Since the level of cellular free radicals may be important for cell survival, we hypothesized that the increase of MnSOD in the mitochondria of mesothelioma cells may alter the free radical levels of these organelles. First, MnSOD activity was compared to the activities of two constitutive mitochondrial enzymes; MnSOD activity was 20 times higher in the mesothelioma cells than in the mesothelial cells, whereas the activities of citrate synthase and cytochrome *c* oxidase did not differ significantly in the two cell lines. This indicates that the activity of MnSOD per mitochondrion was increased in the mesothelioma cells. Superoxide production was assayed in the isolated mitochondria of these cells using lucigenin chemiluminescence. Mitochondrial superoxide levels were significantly lower (72%) in the mesothelioma cells compared to the mesothelial cells. Oxidant production in intact cells, assayed by fluorimetry using 2′,7′-dichlorodihydrofluorescein as a fluorescent probe, did not differ significantly between these cells. We conclude that mitochondrial superoxide levels are lower in mesothelioma cells compared to nonmalignant mesothelial cells, and that this difference may be explained by higher MnSOD activity in the mitochondria of these cells. Oxidant production was not different in these cells, which may be due to the previously observed increase in H_2_O_2_-scavenging mechanisms of mesothelioma cells. © 1999 Cancer Research Campaign


*British Journal of Cancer* (1999) **80**(1/2), 25–31
© 1999 Cancer Research Campaign
Article no. bjoc.1998.0316

### Generation of reactive oxygen species by human mesothelioma cells

**K Kahlos[1], S Pitkänen[3], I Hassinen[2], K Linnainmaa[4] and VL Kinnula[1]**

Departments of [1]Internal Medicine and [2]Medical Biochemistry, University of Oulu, Kajaanintie 50A, FIN-90220 Oulu, Finland; [3]Department of Dermatology, University of Helsinki, Meilahdentie 2, FIN-00250, Helsinki, Finland; [4]Finnish Institute of Occupational Health, Topeliuksenkatu 41A, FIN-00250 Helsinki, Finland

**Summary** Malignant mesothelioma cells contain elevated levels of manganese superoxide dismutase (MnSOD) and are highly resistant to oxidants compared to non-malignant mesothelial cells. Since the level of cellular free radicals may be important for cell survival, we hypothesized that the increase of MnSOD in the mitochondria of mesothelioma cells may alter the free radical levels of these organelles. First, MnSOD activity was compared to the activities of two constitutive mitochondrial enzymes; MnSOD activity was 20 times higher in the mesothelioma cells than in the mesothelial cells, whereas the activities of citrate synthase and cytochrome *c* oxidase did not differ significantly in the two cell lines. This indicates that the activity of MnSOD per mitochondrion was increased in the mesothelioma cells. Superoxide production was assayed in the isolated mitochondria of these cells using lucigenin chemiluminescence. Mitochondrial superoxide levels were significantly lower (72%) in the mesothelioma cells compared to the mesothelial cells. Oxidant production in intact cells, assayed by fluorimetry using 2′,7′-dichlorodihydrofluorescein as a fluorescent probe, did not differ significantly between these cells. We conclude that mitochondrial superoxide levels are lower in mesothelioma cells compared to nonmalignant mesothelial cells, and that this difference may be explained by higher MnSOD activity in the mitochondria of these cells. Oxidant production was not different in these cells, which may be due to the previously observed increase in $H_2O_2$-scavenging mechanisms of mesothelioma cells.

**Keywords:** mesothelioma; reactive oxygen species; oxidant; antioxidant; superoxide dismutase; mitochondria

Tumour cell lines generate reactive oxygen species (ROS) superoxide and hydrogen peroxide ($H_2O_2$), and this generation, if also occurring in vivo, might have effects on tumour proliferation and drug resistance (Szatrowski and Nathan, 1991; Burdon, 1995). ROS production has also been suggested to have an important role in mitochondrial control of cell survival and apoptosis (Burdon, 1997; Kroemer et al, 1997). Manganese superoxide dismutase (MnSOD), a mitochondrial antioxidant enzyme, dismutates superoxide to hydrogen peroxide and oxygen (Fridovich, 1975) and is induced by several cytokines (Wong and Goeddel, 1988; Hirose et al, 1993), changes in the cellular redox state (Warner et al, 1996) and asbestos fibres (Janssen et al, 1992). MnSOD eliminates mitochondrial superoxide radicals and may also contribute to the cell survival of non-malignant and malignant cells. In fact, it has been reported recently that MnSOD may be anti-apoptotic (Slater et al, 1995; Keller et al, 1998; Manna et al, 1998).

Human pleural mesothelioma, which originates from mesothelial cells, is a fatal tumour associated with occupational exposure to asbestos fibres. Its pathogenesis has been postulated to be associated with the generation of free radicals (Kamp et al, 1992; Mossman et al, 1996). In contrast to previous observations, which have shown that MnSOD is usually low in malignant tumours (Oberley and Buettner, 1979; Oberley and Oberley, 1997), we have recently shown that the mRNA level, immunoreactive protein and specific activity of MnSOD are highly expressed in the tumour cells of malignant mesotheliomas when compared to healthy pleura or non-malignant transformed human mesothelial cells (Met5A) (Kinnula et al, 1996; Kahlos et al, 1998). Mesothelioma is resistant to cytotoxic drugs and radiation, both of which are known to induce generation of ROS (Sinha and Mimnaugh, 1990; Nakano et al, 1996). Accordingly, mesothelioma cell line cells are very resistant to a superoxide radical-producing oxidant, menadione, and to epirubicin, which is an anthracycline often used in the treatment of mesothelioma (Kinnula et al, 1996; Kahlos et al, 1998).

We hypothesize that the increase of MnSOD in mesothelioma cells may affect ROS levels in the mitochondria or cytosolic compartments of these cells. To verify that high MnSOD in mesothelioma cells is related to an increased level of this enzyme in the mitochondrial compartment, and not to an increased number or volume of mitochondria per cell, the activities of two constitutive mitochondrial enzymes, citrate synthase and cytochrome *c* oxidase, were measured. In order to test the hypothesis that superoxide levels in these cells with different MnSOD activities may be altered, superoxide production was assayed in isolated mitochondria of malignant mesothelioma cell line cells (M38K, high MnSOD level) and of non-malignant mesothelial cell line cells (Met5A, low MnSOD level). Then, in order to investigate whether the altered level of superoxide at the mitochondria would lead to an increase in $H_2O_2$ formation and to increased oxidant stress in these cells, peroxide formation by these cells was estimated with a fluorimetric assay using 2′,7′ -dichlorodihydrofluorescein as a probe. To

*Received 13 July 1998*
*Revised 1 October 1998*
*Accepted 29 October 1998*

*Correspondence to:* K Kahlos further compare ROS generation in these non-malignant and malignant cells, additional experiments were conducted in cells pre-treated either with aminotriazole (ATZ) to inhibit catalase or buthionine sulfoximine (BSO) to deplete glutathione by inhibiting the rate-limiting enzyme in glutathione synthesis, γ-glutamylcysteine synthetase (γ-GCS). These pre-treated cells were exposed to menadione, which is a quinone known to generate free radicals intracellularly (Powis, 1989).

### METHODS

### Cell culture

Continuous mesothelioma cell line cells (M38K) along with five other mesothelioma cell lines were originally established and characterized from tumours of untreated mesothelioma patients (Pelin-Enlund et al, 1990). M38K cells were chosen because they contain the highest MnSOD activity of these cell lines and are extremely resistant to oxidant-induced cytotoxicity (Kinnula et al, 1996, 1998; Kahlos et al, 1998). Transformed human pleural mesothelial cells (Met5A) were SV-40 virus transformed, immortalized, non-tumorigenic and near-diploid cells (Ke et al, 1989), and were a gift from the National Cancer Institute, Bethesda, MD, USA (Dr CC Harris). Both cell types were cultured on uncoated plastic T25 flasks (Nunclon, Nalge Nunc International, Denmark) or on 24-well microtiter plates (Falcon Multiwell 3047, Becton Dickinson Labware, NJ, USA) in RPMI-1640 medium supplemented with heat-inactivated 10% fetal calf serum, 0.03% L-glutamine, and the antibiotics streptomycin and penicillin (all from LTI Life Technologies, Paisley, UK) at 37°C in 5% carbon dioxide atmosphere.

### Enzyme activities

All enzyme activities were measured by spectrophotometric methods from cells disrupted with 1% Triton X-100. Total superoxide dismutase activity was measured according to the method of Crapo et al (1978). The activity was assessed following the decrease in the rate of reduction (the rate of change in absorbancy at 550 nm) of 12.8 μM cytochrome *c* (Sigma, St Louis, MO, USA) in the presence of 0.5 mM xanthine (Sigma) and xanthine oxidase (Boehringer Mannheim, Germany). MnSOD activity was distinguished from copper–zinc superoxide dismutase (CuZnSOD) activity by its resistance to 1 mM potassium cyanide (Riedel-de-Haen, Seetze, Germany). The activity of citrate synthase, a marker enzyme for the mitochondrial matrix, was assessed according to the method of Shepherd and Garland (1969). The cell sample was added to the reaction solution containing 10 mM L-malate, 41 μg $ml^{-1}$ malate dehydrogenase and 0.5 mM nicotinamide–adenine dinucleotide (NAD) (Sigma), and the reaction was initiated with 0.1 mM acetyl–CoA (coenzyme A). Citrate synthase activity was measured by following the rate of reduction of NAD (Shepherd and Garland, 1969). As a marker enzyme for the mitochondrial inner membrane cytochrome *c* oxidase activity was assayed by the method of Wharton and Tzagoloff (1969). The rate of oxidation of 30 μM reduced cytochrome *c* in 50 mM potassium phosphate buffer was measured by following the decrease in the absorbancy of its α band at 550 nm (Wharton and Tzagoloff, 1969). The protein concentration was measured according to the method of Lowry and co-workers (1951).

### Generation of superoxide by isolated mitochondria

A chemiluminescent probe, lucigenin, was employed to measure superoxide production by isolated mitochondria with a luminometer (1253 Luminometer, Bio-Orbit, Finland). The mitochondria were isolated as described by Pitkänen et al (1996). Frozen-thawed mitochondria (15 μg of mitochondrial protein) was added to a solution of 10 mM $K_2HPO_4$, pH 10.5, and the cuvette was placed into the counter. Background was subtracted and counting was initiated by adding the reduced form of NAD (NADH) (Sigma, 50 μg in 10 μl water) (Liochev and Fridovich, 1997) as a substrate for the respiratory chain electron transport. The luminometer indicates the rates of superoxide production as counts per second measured after 30 s when the steady state has been reached. These units were converted to nmol $min^{-1}$ $mg^{-1}$ of mitochondrial protein using a standard curve described by Pitkänen and Robinson (1996).

### Oxidant production by intact cells

Generation of $H_2O_2$ and other peroxides was measured using a fluorimetric microplate assay established by Rosenkranz et al (1992). A fluorescent probe, 2′,7′-dichlorodihydrofluorescein-diacetate (DCDHF-DA) (Molecular Probes, OR, USA), diffuses to the cell, hydrolyses to 2′,7′-dichlorodihydrofluorescein and is oxidized to the highly fluorescent compound 2′,7′ -dichlorofluorescein in the presence of intracellular $H_2O_2$ and hydroperoxides (Bass et al, 1983; Cathcart et al, 1983). The cells, cultured on 24-well microtiter plates (80 000 cells per well) to near-confluency (2–3 days), were incubated for 30 min with 5 μM DCDHF-DA. The cultures were then washed with phosphate-buffered saline (PBS), and the intensity of fluorescence was measured by a spectrofluorimeter (excitation wavelength 485 nm, emission wavelength 535 nm) capable of reading microtiter plates (1420 Victor multilabel counter, Wallac, Inc., Turku, Finland). The fluoresence intensity was followed every 10 min for 120 min, and each experiment was done in quadruplicate at the time. The background (the fluorescence of cells without DCDHF-DA pre-labelling) was subtracted from the results. A similar initial amount of cells was co-cultured in equal conditions for each experiment, and at the time of the fluorimetric analysis the cell protein content was assessed from these co-cultured plates according to the Bio-Rad method (Bio-Rad, Hercules, CA, USA) (Bradford, 1976). The final results are expressed as fluorescence intensity per cell protein (means of three independent experiments ± s.d.).

### Pre-treatment with aminotriazole or buthionine sulfoximine

The cells were pre-treated either with ATZ (Sigma, 30 mM for 60 min) to inactivate catalase, or with BSO (Sigma, 0.2 mM for 15 h) to inhibit γ-GCS in order to cause glutathione depletion. The concentrations and effects of these inhibitors have been reported in earlier investigations (Margoliash et al, 1960; Buckley et al, 1991; Kinnula et al, 1992*a*, 1998). These concentrations are not toxic, and they have caused at least 85% inhibition of catalase and depletion of glutathione in these same cells (Kinnula et al, 1992*a*). After the pre-treatments the cells were washed and incubated with 5 μM DCDHF-DA for 30 min, washed and measured for fluorescence.

© Cancer Research Campaign 1999

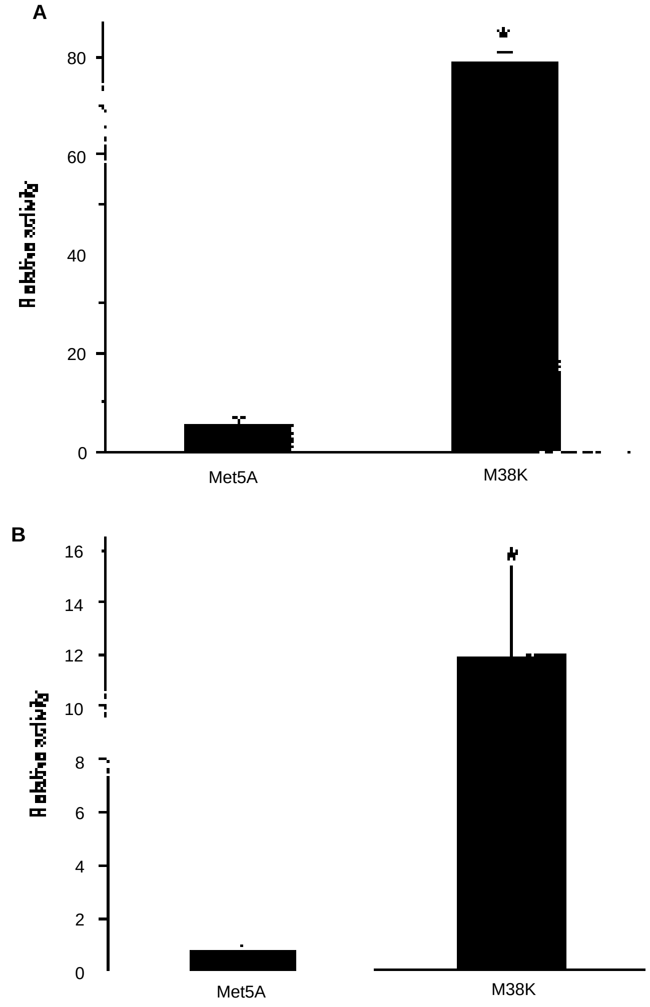

**Figure 1** The ratio of MnSOD activity to the activities of citrate synthase (**A**) and cytochrome *c* oxidase **(B)** in non-malignant mesothelial Met5A cells and mesothelioma M38K cells.* $P < 0.05$ compared to Met5A cells ($n = 3$)

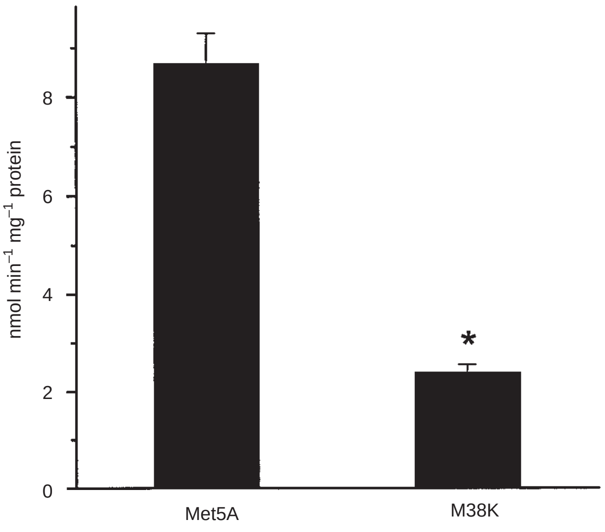

**Figure 2** Superoxide production in isolated mitochondria of Met5A mesothelial cells and M38K mesothelioma cells.* $P < 0.05$ compared to Met5A cells ($n = 3$)

### Oxidant exposure

Washed cells prelabelled with DCDHF-DA were exposed to a superoxide producing oxidant, menadione (10 μM in PBS), and measured immediately for fluorescence. This concentration was chosen because our previous studies have shown it to be marginally toxic to these cells in these incubation conditions in vitro (Kahlos et al, 1998).

### Statistical analysis

The results are expressed as mean ± s.d. of three to seven experiments. Two groups were compared using a two-tailed Student's *t*-test and multiple groups were compared using analysis of variance and Scheffe's post hoc test. $P < 0.05$ was considered significant.

### RESULTS

### MnSOD, citrate synthase and cytochrome *c* oxidase

Previous studies on alveolar type II cells of hyperoxic rats and human renal carcinoma cells of granular type have shown that the elevated level of MnSOD in these cells is at least partly explained by increased volume of mitochondria per cell (Oberley et al, 1994; Vincent et al, 1994). The results of the present study confirm our earlier finding on elevated MnSOD activity in M38K cells as compared to Met5A cells (Kinnula et al, 1996), the specific activity of MnSOD being 36.0 ± 3.4 U $mg^{-1}$ protein in M38K cells and 1.8 ± 1.0 U $mg^{-1}$ protein in Met5A cells. Moreover, the activities of the mitochondrial enzymes citrate synthase and cytochrome *c* oxidase, which have been used as constitutive mitochondrial enzymes (Freeman et al, 1986; Oberley et al, 1987; Vuorinen et al, 1995), did not differ significantly between the two cell lines. The activity of citrate synthase was 45.9 ± 5.3 nmol $min^{-1}$ $mg^{-1}$ protein in M38K cells and 32.7 ± 10.7 nmol $min^{-1}$ $mg^{-1}$ protein in Met5A cells ($P = 0.127$), the corresponding activities for cytochrome *c* oxidase being 3.2 ± 1.0 and 2.4 ± 0.9 nmol $min^{-1}$ $mg^{-1}$ protein ($P = 0.340$). The ratios of MnSOD to these enzymes in these two cell lines are presented in Figure 1, and they show the increase of MnSOD activity to be due to the elevated level of the enzyme per mitochondria.

### Mitochondrial superoxide production

Superoxide generation in the mitochondrial compartment of M38K mesothelioma cells and Met5A mesothelial cells was assessed by lucigenin chemiluminescence. Very small amounts of superoxide were generated before respiratory chain was initiated (i.e. background which was subtracted). Isolated mitochondria of M38K cells generated significantly less superoxide than Met5A cells (0.1 ± 0.01 vs 0.37 ± 0.03 counts per s for every 15 μg of mitochondrial protein respectively; $P = 0.00007$). Figure 2 shows mitochondrial superoxide generation standardized with xanthine oxidase (Pitkänen and Robinson, 1996).

### Oxidant production by intact cells

To further assess ROS generation in these cells, DCDHF-DA fluorescence was used to follow oxidant generation in intact cells and in cells that had been pre-treated with ATZ or BSO to inhibit catalase or to cause glutathione depletion respectively. When

© Cancer Research Campaign 1999

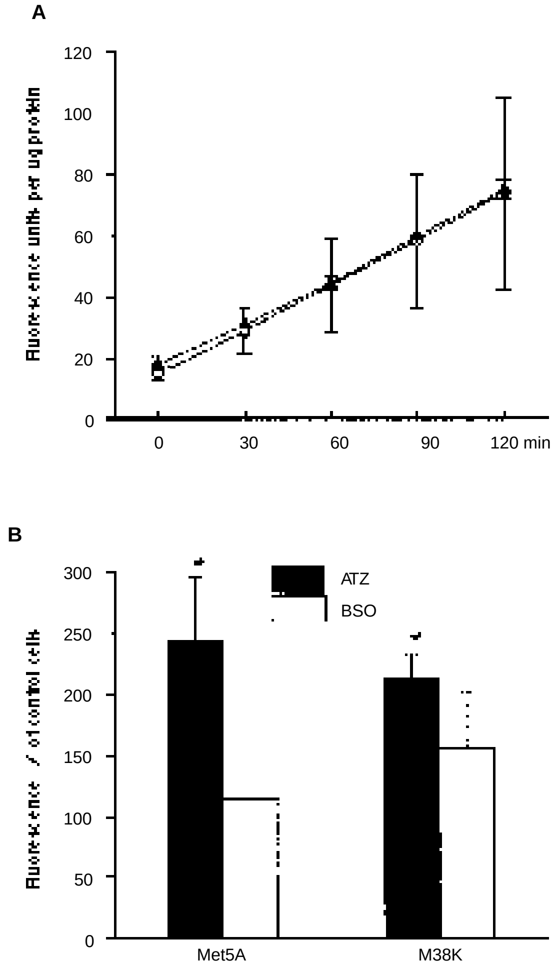

**Figure 3** **(A)** Oxidant production by intact mesothelioma M38K cells (▲) and mesothelial Met5A cells (O) using DCDHF-DA as a fluorescent probe. The results are expressed as fluorescence units per μg of protein per well at different time points during the first 120 min incubation (mean ± s.d. of three individual experiments done in quadruplicate). **(B)** The effect of catalase inhibition by aminotriazole (ATZ) pre-treatment and GSH depletion by buthionine sulfoximine (BSO) pre-treatment on $H_2O_2$ generation by Met5A and M38K cells. The results are presented as the percentage of the fluorescence of untreated control cells. * $P < 0.05$ compared to control cells ($n = 3$). No significant differences were found between the two cell types

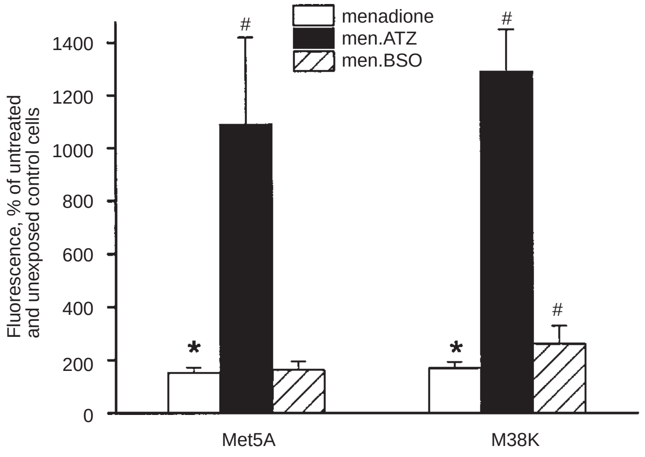

**Figure 4** The relative DCDHF-mediated fluorescence of Met5A cells and M38K cells exposed to menadione (10 μM) with pre-treatments with either aminotriazole or buthionine sulfoximine (menad. = menadione exposure without pre-treatment; menad.ATZ = menadione exposure on cells pre-treated with aminotriazole; menad. BSO = menadione exposure on cells pre-treated with buthionine sulfoximine). The results are presented as the percentage of the fluorescence of unexposed and untreated control cells (means of three to seven experiments each done in quadruplicate ± s.d.). * $P < 0.05$ compared to unexposed control cells, # $P < 0.05$ compared to menadione-exposed cells standardized against the cell protein, the fluorescence intensity did not differ significantly between M38K and Met5A cells (Figure 3A). In particular, catalase inhibition caused a potent increase in the fluorescence of M38K cells and Met5A cells, the enhancement being 213% in M38K cells and 244% in Met5A cells. On the other hand, glutathione depletion did not cause a significant change in the fluorescence of these cells (Figure 3B).

Additional experiments were conducted to assess the effects of exogenous oxidant on the ROS generation of these cells. Menadione (10 μM) caused 68% and 59% increases in the fluorescence in M38K and Met5A cells, respectively, and this response was parallel in the two cell types. The fluorescence in menadione-exposed cells was markedly enhanced by ATZ pre-treatment in both cell types, while the enhancement by BSO pre-treatment was less intense and only significant in M38K cells (Figure 4).

### DISCUSSION

Although many studies have shown that MnSOD is lower in malignant tissue and malignant cells than in their non-malignant counterparts (Sun, 1990), MnSOD has been shown to be high in human thyroid tumours (Nishida et al, 1993), human renal adenocarcinoma of granular cell type (Oberley et al, 1994), human brain tumours (Cobbs et al, 1996) and human malignant mesothelioma (Kahlos et al, 1998) as compared to corresponding non-malignant control tissues. The mesothelioma tumour biopsies and cell lines used in our studies are from untreated mesothelioma patients. Mesothelioma is a tumour which arises from the malignant transformation of mesothelial cells. Met5A cells, though not primary mesothelial cells, are considered representative in vitro models for examining the biology of non-malignant mesothelial cells (Ke et al, 1989).

MnSOD is coded by a nuclear gene and, therefore, is translated in the extramitochondrial compartment. The translation product is a precursor which contains a mitochondrial targeting sequence so that practically all enzyme is found in the mitochondrion (Shimoda-Matsubayashi et al, 1996). The elevation of MnSOD in cells may be associated either with an increased level of this enzyme in the mitochondria or with an increased number of mitochondria per cell. In fact, an elevated expression of MnSOD in human renal carcinoma of granular cell type (Oberley et al, 1994) and in alveolar type II cells of hyperoxic rats (Vincent et al, 1994) was associated not only with increased MnSOD expression per mitochondrion, but also with an increased number or volume of mitochondria per cell. Cytochrome *c* oxidase, a mitochondrial inner membrane enzyme, is known to be stable, and it has been used as a mitochondrial marker enzyme (Vuorinen et al, 1995), whereas citrate synthase is a marker for the mitochondrial matrix. When compared to the activities of these enzymes, MnSOD activity was sixfold higher in mesothelioma M38K cells compared to Met5A cells, reflecting markedly enhanced MnSOD activity per mitochondrion.

Although lucigenin chemiluminescence is not specific to superoxide alone, the use of lucigenin permits a rough estimation of the

© Cancer Research Campaign 1999

rate of superoxide production (Pitkänen and Robinson, 1996). The method does not measure total superoxide generation but its net appearance which is the difference between its total production and scavenging. This study showed that the mitochondrial superoxide levels of malignant M38K cells with their higher MnSOD activity per mitochondrion were 72% less than that of non-malignant Met5A cells. Thus, the observed level of superoxide in mitochondrial preparations seemed to be highly dependent on the level of active MnSOD. Such a correlation between mitochondrial superoxide level and MnSOD has been previously shown in cultured human fibroblasts (Pitkänen and Robinson, 1996). In one previous study, the mitochondria of rat hepatoma cells were shown to have low MnSOD activity, which was associated with normal mitochondrial superoxide generation (Bize et al, 1980). No studies are available on mitochondrial superoxide production in tumours expressing high mitochondrial MnSOD.

Mitochondrial free radical generation may have a significant effect on the cellular redox state, cell proliferation and apoptosis, which are important features in cancer biology (Slater et al, 1995; Burdon, 1997). ROS and changes in the redox state of the cell can initiate reactions leading to the disruption of mitochondrial membrane, depletion of high-energy nucleotides, disturbance of calcium homeostasis and activation of genes, which may result in cell death (Kroemer, 1997). In fact, our recent study showed that M38K cells are much more resistant than Met5A cells in maintaining their cellular energy state, ATP level and cell survival (Kahlos et al, 1998).

Although MnSOD dismutates superoxide to oxygen and $H_2O_2$, which is freely diffusible, increased superoxide elimination in the mitochondria did not lead to increased $H_2O_2$ production in intact mesothelioma cells. One explanation could be that these mesothelioma cells express higher levels of $H_2O_2$-scavenging enzymes and glutathione (Kahlos et al, 1998; Kinnula et al, 1998). On the other hand, the generation of ROS in intact cells is not necessarily parallel with the free radical generation of the mitochondria. ROS can also be scavenged by mitochondrial $H_2O_2$-scavenging mechanisms (Radi et al, 1991). They are also generated and scavenged in other parts of the cell. DCDHF-DA, which was used for the detection of ROS in intact cells, diffuses across the cell membrane and incorporates into the hydrophobic lipid regions in the cell (Bass et al, 1983). Although this method is widely used to measure $H_2O_2$ production, it detects also various peroxides (Royall and Ischiropoulos, 1993). Since the probe can theoretically move from one area to another both inside and outside the cell, its localization does not indicate the precise compartment, where the original reaction has occurred. In spite of the fact that DCDHF-DA has been shown to detect intracellular ROS generation, the assay system can also detect fluorescent probe leaked through the cell membrane or ROS generated on the plasma membrane (Royall and Ischiropoulos, 1993). In our system, the assay was calibrated to measure the fluorescence on the bottom of each well, because our cells are adherent. Furthermore, the volume of the assay medium was kept as small as possible to minimize the effects caused by dilution. Based on these assumptions, we can conclude that the net generation of $H_2O_2$ and other peroxides was very similar in malignant M38K cells and non-malignant Met5A cells.

Antioxidants, such as superoxide dismutases, may be anti-apoptotic (Troy and Shelanski, 1994; Keller et al, 1998; Manna et al, 1998). The role of MnSOD in this issue is, however, complicated because there is powerful evidence to suggest that MnSOD is antiproliferative in malignant cells (Oberley and Oberley, 1997). It has been shown that transfection of the MnSOD gene leads to a suppression of the malignant phenotype and decreased cell proliferation. The situation is more complicated in vivo, where simultaneous induction of multiple antioxidant enzymes is possible (Tew, 1994; Kahlos et al, 1998; Kinnula et al, 1998). The same is true of M38K cells, which represent an immortal cell line established from one of our mesothelioma patients. In fact, M38K mesothelioma cells are not only high in MnSOD, but also have higher catalase, glutathione-S-transferase and glutathione contents than Met5A mesothelial cells (Kahlos et al, 1998; Kinnula et al, 1998).

The relative role of various antioxidants in scavenging $H_2O_2$ may vary considerably in different cell types. Previous studies have indicated that catalase activity is high in alveolar pneumocytes and macrophages, and catalase inhibition significantly reduces $H_2O_2$ consumption by these cells (Kinnula et al, 1992*b*; Pietarinen et al, 1995). On the other hand, endothelial cells appear to protect themselves by the glutathione redox cycle, but not by catalase (Schraufstätter et al, 1985; Andreoli et al, 1992; Kinnula et al, 1992*c*). To further examine which antioxidant enzyme mechanism plays a major role as a free radical scavenger in these cells, we inhibited catalase with ATZ or γ-GCS with BSO and measured oxidant generation in both intact unexposed cells and cells exposed to menadione. Both unexposed and exposed cells generated significantly more $H_2O_2$ if catalase had been inhibited than the cells in which γ-GCS had been inhibited. This effect was similar in non-malignant mesothelial and malignant mesothelioma cells, which also suggests that other undefined mechanisms contribute to the oxidant generation in these cells.

In conclusion, human mesothelioma cells contain highly elevated MnSOD levels and simultaneously decreased generation of superoxide in the mitochondrial compartment of these cells. This phenomenon, if also occurring in vivo, may prolong cell survival and contribute to the oxidant resistance of these cells. The production of $H_2O_2$ or other peroxides by intact mesothelioma cells did not differ between non-malignant mesothelial cells and malignant mesothelioma cells, possibly due to the previously observed effective $H_2O_2$-scavenging capacity of these mesothelioma cells. The effects of the lowered free radical generation on the cell survival and drug resistance of these cells remains to be investigated.

### ACKNOWLEDGEMENTS

The authors thank Dr CC Harris for providing the Met5A cells and Mrs Raija Sirviö for her skillful assistance. This work was financially supported by the University of Oulu, the Cancer Society of Northern Finland, the Finnish Anti-Tuberculosis Association Foundation, the Emil Aaltonen Foundation and Finnish Cultural Foundation.

### REFERENCES

Andreoli SP, Mallet CC, McAteer JA and Williams LV (1992) Antioxidant defense mechanisms of endothelial cells and renal tubular epithelial cells in vitro: role of the glutathione redox cycle and catalase. *Pediatr Res* **32**: 360–365

Bass DA, Parce JW, Dechatelet LR, Szejda P, Seeds MC and Thomas M (1983) Flow cytometric studies of oxidative product formation by neutrophils: a graded response to membrane stimulation. *J Immunol* **130**: 1910–1917

© Cancer Research Campaign 1999

Bize IB, Oberley LW and Morris HP (1980) Superoxide dismutase and superoxide in Morris hepatomas. *Cancer Res* **40**: 3686–3693
Bradford M (1976) A rapid and sensitive method for the quantitation of microgram quantities of protein utilizing the principal of protein-dye binding. *Anal Biochem* **72**: 248–254
Buckley BJ, Kent RS and Whorton R (1991) Regulation of endothelial cell prostaglandin synthesis by glutathione. *J Biol Chem* **266**: 16659–16666
Burdon RH (1995) Superoxide and hydrogen peroxide in relation to mammalian cell proliferation. *Free Rad Biol Med* **18**: 775–794
Burdon RH (1997) Oxyradicals as signal transducers. In *Oxygen Stress and Signal Transduction*, Forman HJ and Cadenas E (eds), pp 289–319. Chapman & Hall: New York
Cathcart R, Schwiers E and Ames BN (1983) Detection of picomole levels of hydroperoxides using a fluorescent dichlorofluorescein assay. *Anal Biochem* **134**: 111–116
Cobbs CS, Levi DS, Aldape K and Israel MA (1996) Manganese superoxide dismutase expression in human central nervous system tumors. *Cancer Res* **56**: 3192–3195
Crapo JD, McCord JM and Fridovich I (1978) Preparation and assay of superoxide dismutases. *Methods Enzymol* **53**: 382–393
Freeman BA, Mason RJ, Williams MC and Crapo JD (1986) Antioxidant enzyme activity in alveolar type II cells after exposure of rats to hyperoxia. *Exp Lung Res* **10**: 203–222
Fridovich I (1975) Superoxide dismutases. *Biochim Biophys Acta* **877**: 147–159
Hirose K, Longo DL, Oppenheim JJ and Matsushima K (1993) Overexpression of mitochondrial manganese superoxide dismutase promotes survival of tumor cells exposed to IL-1, tumor necrosis factors, selected anticancer drugs and ionizing radiation. *FASEB J* **7**: 361–368
Janssen YMW, Marsh JP, Absher MP, Hemenway D, Vacek PM, Leslie KO, Borm PJA and Mossman BT (1992) Expression of antioxidant enzymes in rat lungs after inhalation of asbestos or silica. *J Biol Chem* **267**: 10623–10630
Kahlos K, Anttila S, Asikainen T, Kinnula K, Raivio KO, Mattson K, Linnainmaa K and Kinnula VL (1998) Manganese superoxide dismutase in healthy human pleural mesothelium and in malignant pleural mesothelioma. *Am J Respir Cell Mol Biol* **18**: 570–580
Kamp DW, Graceffa P, Pryor WA and Weitzman SA (1992) The role of free radicals in asbestos-induced diseases. *Free Rad Biol Med* **12**: 293–315
Ke Y, Reddel RR, Gerwin BI, Reddel HK, Somers ANA, McMenamin MG, LaVeck MA, Stahel RA, Lechner JF and Harris CC (1989) Establishment of a human in vitro mesothelial cell model system for investigating mechanisms of asbestos-induced mesothelioma. *Am J Pathol* **134**: 979–991
Keller JN, Kindy MS, Holtsberg FW, St and Clair DK, Yen H-C, Germeyer A, Steiner SM, Bruce-Keller AJ, Hutchins JB and Mattson MP (1998) Mitochondrial manganese superoxide dismutase prevents neural apoptosis and reduces ischemic brain injury: suppression of peroxynitrite production, lipid peroxidation, and mitochondrial dysfunction. *J Neurosci* **18**: 687–697
Kinnula VL, Everitt JI, Mangum JB, Chang LY and Crapo JD (1992*a*) Antioxidant defense mechanisms in cultured pleural mesothelial cells. *Am J Respir Cell Mol Biol* **7**: 95–103
Kinnula VL, Chang L, Everitt JI and Crapo JD (1992*b*) Oxidants and antioxidants in alveolar epithelial type II cells: in situ, freshly isolated, and cultured cells. *Am J Physiol* **262**: L69–L77
Kinnula VL, Whorton AR, Chang L-Y and Crapo JD (1992*c*) Regulation of hydrogen peroxide generation in cultured endothelial cells. *Am J Respir Cell Mol Biol* **6**: 175–182
Kinnula VL, Pietarinen-Runtti P, Raivio KO, Kahlos K, Pelin K, Mattson K and Linnainmaa K (1996) Manganese superoxide dismutase in human pleural mesothelioma cell lines. *Free Rad Biol Med* **21**: 527–532
Kinnula K, Linnainmaa K, Raivio KO and Kinnula VL (1998) Endogenous antioxidant enzymes and glutathione-S-transferase in protection of mesothelioma cells against hydrogen peroxide and epirubicin toxicity. *Br J Cancer* **77**: 1097–1102
Kroemer G (1997) The proto-oncogene Bcl-2 and its role in regulating apoptosis. *Nature Med* **3**: 614–620
Kroemer G, Zamzani N and Susin SA (1997) Mitochondrial control of apoptosis. *Immunol Today* **18**: 44–51
Liochev SI and Fridovich I (1997) Lucigenin (bis-*N*-methylacridinum) as a mediator of superoxide anion production. *Arch Biochem Biophys* **337**: 115–120
Lowry OH, Rosenbrough NR, Farr AL and Randall RJ (1951) Protein measurement with the pholin phenol reagent. *J Biol Chem* **193**: 265–275
Manna SK, Zhang HJ, Yan T, Oberley LW and Aggarwal BB (1998) Overexpression of manganese superoxide dismutase suppresses tumor necrosis factor-induced apoptosis and activation of nuclear transcription factor-kappaB and activated protein-1. *J Biol Chem* **273**: 13245–13254
Margoliash E, Novogrodsky A and Scheijter A (1960) Irreversible reaction of 3-amino 1:2,4-triazole and related inhibitors with the protein catalase. *Biochem J* **74**: 339–348
Mossman BT, Kamp DW and Weitzman SA (1996) Mechanisms of carcinogenesis and clinical features of asbestos-associated cancers. *Cancer Invest* **14**: 466–480
Nakano T, Oka K and Taniguchi N (1996) Manganese superoxide dismutase expression correlates with p53 status and local recurrence of cervical carcinoma treated with radiation therapy. *Cancer Res* **56**: 2771–2775
Nishida S, Akai F, Iwasaki H, Hosokawa K, Kusunoki T, Suzuki N, Taniguchi S, Hashimoto S and Tamura TT (1993) Manganese superoxide dismutase content and localization in human thyroid tumors. *J Pathol* **169**: 341–345
Oberley LW and Buettner GR (1979) Role of superoxide dismutase in cancer: a review. *Cancer Res* **39**: 1141–1149
Oberley LW and Oberley TD (1997) Role of antioxidant enzymes in cancer phenotype. In *Oxygen, Gene Expression and Cellular Function (Lung Biology in Health and Disease*) Vol 105, Clerch LB and Massaro DJ (eds), pp. 279–307. Marcel Dekker Inc.: New York
Oberley LW, St Clair DK, Autor AP and Oberley TD (1987) Increase in manganese superoxide dismutase activity in the mouse heart after x-irradiation. *Arch Biochem Biophys* **254**: 69–80
Oberley TD, Sempf JM, Oberley MJ, McCormick ML, Muse KE and Oberley LW (1994) Immunogold analysis of antioxidant enzymes in human renal cell carcinoma. *Virchows Arch* **424**: 155–164
Pelin-Enlund K, Husgafvel-Pursiainen K, Tammilehto L, Klockars M, Jantunen K, Gerwin BI, Harris CC, Tuomi T, Vanhala E, Mattson K and Linnainmaa K (1990) Asbestos-related malignant mesothelioma: growth, cytology, tumorigenicity and consistent chromosome findings in cell lines from five patients. *Carcinogenesis* **11**: 673–681
Pietarinen P, Raivio KO, Devlin RB, Crapo JD, Chang L-Y and Kinnula VL (1995) Catalase and glutathione reductase protection of human alveolar macrophages during oxidant exposure in vitro. *Am J Respir Cell Mol Biol* **13**: 431–441
Pitkänen S and Robinson BH (1996) Mitochondrial complex I deficiency leads to increased production of superoxide radicals and induction of superoxide dismutase. *J Clin Invest* **98**: 345–351
Pitkänen S, Raha S and Robinson BH (1996) Diagnosis of complex I deficiency in patients with lactic acidemia using skin fibroblast cultures. *Biochem Mol Med* **59**: 134–137
Powis G (1989) Free radical formation by antitumor quinones. *Free Rad Biol Med* **6**: 63–101
Radi R, Turrens JF, Chang LY, Bush KM, Crap JD and Freeman BA (1991) Detection of catalase in rat heart mitochondria. *J Biol Chem* **266**: 22028–22034
Rosenkranz AR, Schmaldienst S, Stuhlmeier KM, Chen W, Knapp W and Zlabinger GJ (1992) A microplate assay for the detection of oxidative products using 2′, 7′-dichlorofluorescin-acetate. *J Immunol Methods* **156**: 39–45
Royall JA and Ischiropoulos S (1993) Evaluation of 2′,7′-dichlorofluorescin and dihydrorhodamine 123 as fluorescent probes for intracellular $H_2O_2$ in cultured endothelial cells. *Arch Biochem Biophys* **302**: 348–355
Schraufstätter IV, Hinshaw DB, Hyslop PA, Spragg RG and Cochrane CG (1985) Glutathione cycle activity and pyridine nucleotide levels in oxidant-induced injury of cells. *J Clin Invest* **76**: 1131–1139
Shepherd D and Garland PB (1969) Citrate synthase from rat liver. In *Methods in Enzymology*, vol 13, Lowenstein JM (ed), pp. 11–16. Academic Press: New York
Shimoda-Matsubayashi S, Matsumine H, Kobayashi T, Nakagawa-Hattori Y, Shimizu Y and Mizuno Y (1996) Structural difomorphism in the targeting sequence in the human manganese superoxide dismutase gene. *Biochem Biophys Res Comm* **226**: 561–565
Sinha BK and Mimnaugh EG (1990) Free radicals and anticancer drug resistance: oxygen free radicals in the mechanisms of drug cytotoxicity and resistance by certain tumors. *Free Rad Biol Med* **8**: 567–581
Slater AFG, Nobel CSI and Orrenius S (1995) The role of intracellular oxidants in apoptosis. *Biochim Biophys Acta* **1271**: 59–62
Sun Y (1990) Free radicals, antioxidant enzymes, and carcinogenesis. *Free Rad Biol Med* **8**: 583–599
Szatrowski TP and Nathan CF (1991) Production of large amounts of hydrogen peroxide by human tumor cells. *Cancer Res* **51**: 794–798
Tew K (1994) Glutathione-associated enzymes in anticancer drug resistance. *Cancer Res* **54**: 4313–4320
Troy CM and Shelanski ML (1994) Down-regulation of copper/zinc superoxide dismutase causes apoptotic death in PC12 neuronal cells. *Proc Natl Acad Sci* **91**: 6384–6387
Warner BB, Chang L-Y, Auten RL, Ho Y-S and Crapo JD (1996) Redox regulation of manganese superoxide dismutase. *Am J Physiol* **271**: L150–L158

 © Cancer Research Campaign 1999

Wharton DC and Tzagoloff A (1969) Cytochrome oxidase from beef heart mitochondria. In *Methods in Enzymology*, Estabrook RW and Pullman ME (eds), vol 10, pp. 245–250. Academic Press: New York

Wong GH and Goeddel DV (1988) Induction of manganese superoxide dismutase by tumor necrosis factor: possible protective mechanism. *Science* **242**: 941–944

Vincent R, Chang L-Y, Slot JW and Crapo JD (1994) Quantitative immunocytochemical analysis of MnSOD in alveolar type II cells of the hyperoxic rat. *Am J Physiol* **267**: L475–481

Vuorinen K, Ylitalo K, Peuhkurinen K, Raatikainen P, Ala-Rämi A and Hassinen I (1995) Mechanisms of ischemic preconditioning in rat myocardium. *Circulation* **91**: 2810–2818

*© Cancer Research Campaign 1999*
